# The C-reactive protein/albumin ratio predicts long-term outcomes of patients with operable non-small cell lung cancer

**DOI:** 10.18632/oncotarget.13053

**Published:** 2016-11-03

**Authors:** Fanrong Zhang, Lisha Ying, >Jiaoyue Jin, >Kaiyan Chen, >Nan Zhang, >Junzhou Wu, Yimin Zhang, Dan Su

**Affiliations:** ^1^ Department of Oncology, The First Clinical Medical College of Wenzhou Medical University, Wenzhou, China; ^2^ Cancer Research Institute, Zhejiang Cancer Hospital & Key Laboratory Diagnosis and Treatment Technology on Thoracic Oncology of Zhejiang Province, Hangzhou, China; ^3^ Department of Clinical Laboratory, Zhejiang Cancer Hospital, Hangzhou, China

**Keywords:** C-reactive protein, albumin, inflammation-based prognostic score, prediction, survival

## Abstract

**Purpose:**

To investigate the association between C-reactive protein/albumin ratio (CAR), an inflammation-based prognostic score, and clinicopathological factors, as well as its association with long-term outcomes in patients with operable non-small cell lung cancer (NSCLC).

**Methods:**

A total of 617 operable NSCLC patients were retrospectively evaluated and the data of preoperative serum CRP and serum albumin was collected. The correlation between the CAR and clinicopathological factors was analyzed using the chi-square test. A Cox proportional hazards regression model was performed to evaluate the association between the CAR and outcome.

**Results:**

The CAR was significantly related to sex, smoking status, BMI, histology type and clinical stage (*p* = 0.05). The patients with characteristic of male, smoker, BMI under 18.5, squamous cell carcinoma or clinical stage III had a high level of CAR. Additionally, elevated CAR indicated a worse outcome, and the patients with higher CAR had 2.02-fold risk for disease progression (95% CI 1.48-2.74, *p* < 0.001) and 2.61-fold risk for death (95 % CI 2.02-3.37, *p* < 0.001). Multivariate analyses showed the similar results after adjusted by clinicopathological factors and another four inflammation-based prognostic scores.

**Conclusions:**

The CAR is a potential independent predictor for disease progression and death in patients with operable NSCLC.

## INTRODUCTION

As the most common malignant tumor in the world, lung cancer remains a leading cause of cancer-related mortality [1]. Non-small cell lung cancer (NSCLC) consists of approximately 85 % of primary lung cancer [2]. In spite of advances in early detection and diverse treatments, the outcomes of NSCLC patients are still poor, with the 5-year overall survival rate being 18.2 % [2]. As such, it is important to identify promising prognostic biomarkers to help tailor the most beneficial treatment for NSCLC patients.

Growing evidence indicates that the systemic inflammatory response substantially contributes to the tumor initiation and progression, and it is related to a poor prognosis in many tumors [3–6]. In the last decade, the clinical and prognostic values of a number of inflammation-based prognostic scores, including Glasgow prognostic score (GPS), modified GPS (mGPS), neutrophil lymphocyte ratio (NLR) and platelet lymphocyte ratio (PLR), have been validated in many types of cancer [4, 7, 8].

Recently, a novel inflammation-based prognostic score, the C-reactive protein/albumin ratio (CRP/Alb ratio, CAR) was reported as an independent prognostic marker for overall survival (OS) in several types of cancer [9–11]. However, the role of the CAR in patients with operable NSCLC has not been evaluated yet to our knowledge. The present study investigated the connection between CAR and clinicopathological factors and explored the long-term prognostic value of the CAR in operable NSCLC patients.

## RESULTS

### Characteristics of patients

A total of 617 patients were enrolled into this study and their clinicopathologic features are summarized in Table 1. The median age of the patients at diagnosis was 60 (range 30-82) years. Among these patients, 71.8 % (443/617) were younger than 65 years, 74.7 % (461/617) were male, 66.5 % (410/617) were smokers, 8.8 % (54/617) had a body mass index (BMI) of less than 18.5 and 26.3 % (162/617) had a BMI of equal or more than 24. Based on the World Health Organization (WHO) classification standard, 49.3 % (304/617) were squamous cell carcinoma, 48.8 % (301/617) were adenocarcinoma, and 1.9 % (12/617) were others. In terms of tumor grade, 48.8 % (301/617) were well differentiated, 42.0 % (259/617) were poorly differentiated, and this information was missing in 57 cases (9.2 %). In accordance with the International Association for the Study of Lung Cancer (IASLC) TNM staging system, 411 (66.6 %) patients were stage I-II, 200 (32.4 %) patients were stage III.

**Table 1:** Clinicopathological characteristics of 617 operable NSCLC patients and their correlations with CAR

Factors	Case, *n* (%)	Case of CAR < 0.424, *n* (%)	Case of CAR ≥ 0.424, *n* (%)	*p* value
**Age, year**				**0.085**
<65	443 (71.8)	361 (73.4)	82 (65.6)	
≥65	174 (28.2)	131 (26.6)	43 (34.4)	
**Sex**				**<0.001**
Male	461 (74.7)	343 (69.7)	118 (94.4)	
Female	156 (25.3)	149 (30.3)	7 (5.6)	
**Smoking**				**<0.001**
Non-smoker	207 (33.5)	190 (38.6)	17 (13.6)	
Former smoker	195 (31.6)	137 (27.8)	58 (46.4)	
Current smoker	215 (34.8)	165 (33.5)	50 (40.0)	
**BMI, kg/m**^2^				**0.001**
<18.5	54 (8.8)	34 (6.9)	20 (16.0)	
≥18.5 to <24.0	401 (65.0)	315 (64.0)	86 (68.8)	
≥24.0 to <28.0	140 (22.7)	124 (25.2)	16 (12.8)	
≥28.0	22 (3.6)	19 (3.9)	3 (2.4)	
**Histology**				**<0.001**
SCC	304 (49.3)	211 (42.9)	93 (74.4)	
Adenocarcinoma	301 (48.8)	273 (55.5)	28 (22.4)	
Others	12 (1.9)	8 (1.6)	4 (3.2)	
**Grade**				**0.673**
Well	301 (48.8)	235 (47.8)	66 (52.8)	
Poor	259 (42.0)	206 (41.9)	53 (42.4)	
Missing	57 (9.2)	51 (10.4)	6 (4.8)	
**Clinical stage**				**<0.001**
I-II	411 (66.6)	344 (69.9)	67 (53.6)	
III	200 (32.4)	142 (28.9)	58 (46.4)	
Missing	6 (1.0)	6 (1.2)	0 (0)	

Bold values were statistically significant (*p* ≤ 0.05).

Abbreviations: *CAR* C-reactive protein/albumin ratio, *BMI* body mass index, *SCC* squamous cell carcinoma

### CAR and its association with clinicopathological factors

The cutoff value of the CAR based on the OS was determined to be 0.424 in this cohort. Then patients were separated into two groups (CAR < 0.424, *n* = 492, 79.7 %; CAR ≥ 0.424, *n* = 125, 20.3 %) according to the cutoff value. Besides, the cutoff values of the NLR and PLR were 2.631 and 158.6.

The association of the CAR with clinicopathological factors is shown in Table 1. The CAR was significantly related to sex, smoking status, BMI, histology type and clinical stage (*p* ≤ 0.05). The patients with characteristic of male, smoker, BMI under 18.5, squamous cell carcinoma or clinical stage III had a high level of CAR.

### Relationship between CAR and survival

The median follow-up time of the 617 patients was 50 (range 1-108) months. During the follow-up period, 257 (41.7 %) patients relapsed, 283 (45.9 %) patients died and 30 (4.9 %) patients lost.

**Figure 1:**
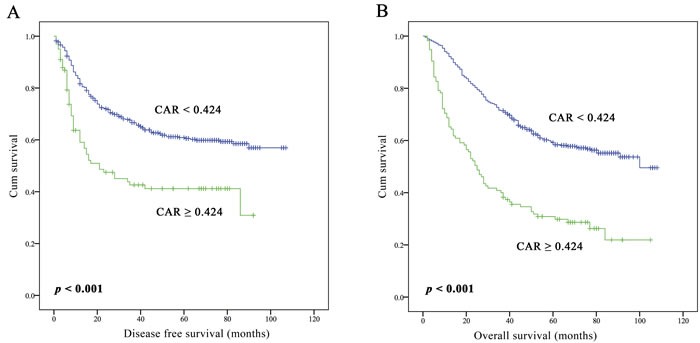
**Kaplan-Meier curves of DFS A. and OS B. according to CAR in 617 operable NSCLC patients**.

As shown in Table 2 and Table 3, the univariate analyses indicated that elevated CAR was correlated with poor disease free survival (DFS) and OS (crude Hazard Ratio [HR] 2.02, 95 % confidence interval [CI] 1.48-2.74, *p* < 0.001 for DFS; and crude HR 2.61, 95% CI 2.02-3.37, *p* < 0.001 for OS). Kaplan-Meier survival curves showed the similar results (Figure 1). Also, CAR was identified to be an independent prognostic factor for the DFS and OS when adjusted by clinicopathological factors and another four inflammation-based prognostic scores. NSCLC patients with high level of the CAR would have worse DFS and OS (adjust HR 1.54, 95% CI 1.10-2.16, *p* = 0.012 for DFS; adjust HR 1.87, 95 % CI 1.41-2.49, *p* < 0.001 for OS) (Table 2, 3).

**Table 2:** Prognostic factors for disease free survival of operable NSCLC patients estimated by univariate and multivariate Cox regression analyses

Factors	Univariate analysis			Multivariate analysis		
	HR	95% CI	*p* value	HR	95% CI	*p* value
**Age, years**						
<65	1					
≥65	1.30	1.00-1.69	0.053			
**Gender**						
Male	1					
Female	0.89	0.67-1.18	0.422			
**Smoking**						
Non-smokers	1		0.829			
Former smokers	1.03	0.76-1.40	0.839			
Current smokers	1.10	0.81-1.48	0.545			
**BMI, kg/m**^2^						
<18.5	1		0.309			
≥18.5 to <24.0	0.81	0.53-1.24	0.323			
≥24.0 to <28.0	0.73	0.45-1.18	0.200			
≥28.0	0.43	0.16-1.12	0.085			
**Histology**						
SCC	1		0.247			
Adenocarcinoma	1.22	0.95-1.57	0.129			
Others	0.78	0.29-2.13	0.632			
**Grade**						
Well	1			1		
Poor	1.58	1.22-2.04	**0.001**	1.63	1.24-2.15	**<0.001**
**Clinical stage**						
I-II	1			1		
III	1.88	1.46-2.43	**<0.001**	1.63	1.23-2.16	**0.001**
**CAR**						
<0.424	1			1		
≥0.424	2.02	1.48-2.74	**<0.001**	1.54	1.10-2.16	**0.012**
**GPS**						
0	1		**<0.001**			
1	1.82	1.37-2.43	**<0.001**			
2	1.36	0.72-2.59	0.345			
**mGPS**						
0	1		**<0.001**			
1	1.92	1.42-2.58	**<0.001**			
2	1.35	0.71-2.56	0.361			
**NLR**						
<2.631	1			1		
≥2.631	1.56	1.21-2.00	**0.001**	1.38	1.04-1.83	**0.026**
**PLR**						
<158.6	1					
≥158.6	1.51	1.16-1.98	**0.003**			

Bold values were statistically significant (*p* ≤ 0.05).

Abbreviations: *HR* hazard ratio, *CI* confidence interval, *BMI* body mass index, *SCC* squamous cell carcinoma, *CAR* C-reactive protein/albumin ratio, *GPS* Glasgow prognostic score, *mGPS* modified Glasgow prognostic score, *NLR* neutrophil lymphocyte ratio, *PLR* platelet lymphocyte ratio

**Table 3:** Prognostic factors for overall survival of operable NSCLC patients estimated by univariate and multivariate Cox regression analyses

Factors	Univariate analysis			Multivariate analysis		
	HR	95% CI	*p* value	HR	95% CI	*p* value
**Age, years**						
<65	1					
≥65	1.36	1.07-1.73	**0.011**			
**Gender**						
Male	1					
Female	0.76	0.58-1.00	**0.049**			
**Smoking**						
Non-smokers	1		0.088			
Former smokers	1.27	0.96-1.68	0.101			
Current smokers	1.35	1.02-1.77	**0.034**			
**BMI, kg/m**^2^						
<18.5	1		0.156			
≥18.5 to <24.0	0.70	0.48-1.01	0.059			
≥24.0 to <28.0	0.63	0.42-0.96	**0.031**			
≥28.0	0.60	0.29-1.18	0.130			
**Histology**						
SCC	1		0.535			
Adenocarcinoma	0.88	0.71-1.12	0.282			
Others	0.83	0.37-1.87	0.652			
**Grade**						
Well	1			1		
Poor	1.33	1.05-1.67	**0.018**	1.31	1.02-1.67	**0.032**
Clinical stage						
I-II	1			1		
III	2.27	1.81-2.86	**<0.001**	1.98	1.54-2.55	**<0.001**
CAR						
<0.424	1			1		
≥0.424	2.61	2.02-3.37	**<0.001**	1.87	1.41-2.49	**<0.001**
**GPS**						
0	1		**<0.001**			
1	1.99	1.54-2.57	**<0.001**			
2	3.79	2.52-5.70	**<0.001**			
**mGPS**						
0	1		**<0.001**			
1	1.92	1.46-2.51	**<0.001**			
2	3.64	2.42-5.46	**<0.001**			
NLR						
<2.631	1			1		
≥2.631	1.86	1.49-2.33	**<0.001**	1.58	1.22-2.04	**0.001**
**PLR**						
<158.6	1					
≥158.6	1.72	1.36-2.18	**<0.001**			

Bold values were statistically significant (*p* ≤ 0.05).

Abbreviations: *HR* hazard ratio, *CI* confidence interval, *BMI* body mass index, *SCC* squamous cell carcinoma, *CAR* C-reactive protein/albumin ratio, *GPS* Glasgow prognostic score, *mGPS* modified Glasgow prognostic score, *NLR* neutrophil lymphocyte ratio, *PLR* platelet lymphocyte ratio

Besides, the multivarite Cox proportional hazards regression analyses demonstrated that NLR, clinical stage and grade were independent predictors for both DFS and OS. NSCLC patients with a high level of NLR were more likely to have worse outcomes than those with a low level of NLR (adjust HR 1.38, 95% CI 1.04-1.83, *p* = 0.026 for DFS; adjust HR 1.58, 95 % CI 1.22-2.04, *p* = 0.001 for OS). Compared with stage I-II NSCLC patients, stage III NSCLC patients were prone to have shorter DFS and OS (adjust HR 1.63, 95% CI 1.23-2.16, *p* = 0.001 for DFS; adjust HR 1.98, 95 % CI 1.54-2.55, *p* < 0.001 for OS). The risks of disease progression and death for NSCLC patients with poor-differentiated tumors were 1.63-fold and 1.31-fold higher than those with well-differentiated tumors, respectively (95% CI 1.24-2.15, *p* < 0.001 for DFS; 95 % CI 1.02-1.67, *p* = 0.032 for OS) (Tables 2, 3).

## DISCUSSION

In the present study, the CAR was significantly related to sex, smoking experience, BMI, histology and clinical stage. Furthermore, it was an independent predictor of disease progression and death for operable NSCLC patients.

Being one of the inflammation-based prognostic scores, the CAR was shown correlative to prognosis in septic patients earlier [12], and it was found useful for predicting OS in hepatocellular carcinoma [9], colorectal cancer [10] and esophageal squamous cell carcinoma [11] recently, which was similar to our result that inflammation had close relationship with tumor progression in operable NSCLC patients. It has been reported that inflammation could facilitate cancer development by means of arousing genomic destabilization, promoting proliferative and survival signaling, inducing invasion and metastasis, subverting immune reaction and altering responses to chemotherapeutic agents [6, 13, 14], which might be the preliminary mechanisms underlying the association between inflammation and cancer outcome.

Besides, the CAR, which consists of serum CRP and serum albumin, may indicate not only inflammatory condition but also nutritional status of cancer patients. The relation between inflammation and nutrition had been demonstrated by many studies and supplement of some nutrient factors could decrease the CAR, reduce inflammation and improve immune function [15–17]. As a matter of fact, this study found that a higher level of CAR was related to a lower BMI level (Table 1). Therefore, the CAR could be used to evaluate the trophic state complementally and guide nutrition improvement treatment, and the practicability needs assessment in clinical practice.

Just like the CAR, the GPS and the mGPS were also calculated by means of serum CRP and serum albumin. As inflammation-based prognostic scores, the GPS and the mGPS had been proved of independent prognostic value in patients with operable cancer or inoperable cancer, as well as cancer population receiving chemo/radiotherapy so far [18]. Nevertheless, the multivariate analysis took the CAR as an independent prognostic factor when both the GPS and the mGPS were confounding factors (Table 2, 3). What's more, there is a fundamental difference that the CAR is quantitative, while the GPS and the mGPS are rather more qualitative. As such, the CAR may enable a better prospect for predicting prognosis and tailoring treatment in malignant tumors.

Furthermore, the multivariate analysis in this study also identified three independent prognostic factors for both DFS and OS in NSCLC patients: clinical stage, grade and NLR (Table 2, 3). It has been generally admitted that cancer patients with inferior clinical stage and grade would have a worse ending. However, with regard to their application value, the CAR would be better than clinical stage and grade in a way because the CAR could be measured before surgery while only after surgery could clinical stage and grade be ultimately confirmed. As for NLR, it had been found to have correlation with poor survival in many types of cancers [19–21], a result consistent with the present study. The interrelation between elevated levels of NLR and poor outcomes of tumors may be explained as an increase in neutrophils or decrease in lymphocytes that may produce cytokines, restrain lymphokine-activated killer cells and facilitate the progression of cancer [21–23]. Composed of different indices, the CAR and the NLR could be mutually supplemented when used in clinical.

To sum up, on the basis of two simple, objective, conventional, and inexpensive laboratory indices, the CAR was a potential prognostic predictor of both DFS and OS in patients with operable NSCLC and enables a bright prospect in clinical practice. However, our patients in this study were from a single institution, thus, the representativeness of our sample is limited and it remains to be validated in large-scale prospective researches.

## MATERIALS AND METHODS

### Patients

A total of 617 primary NSCLC patients who underwent surgery in Zhejiang Cancer Hospital between November 2006 and December 2009 were retrospectively enrolled. Surgeries were operated by experienced surgeons of Zhejiang Cancer Hospital and all patients were histologically confirmed to have NSCLC. Patients with infection or other inflammatory conditions were excluded from this study. Most patients had complete clinicopathological data including age, sex, smoking status, BMI, histological type, tumor grade and clinical stage. This study was approved by the Institutional Review Board of Zhejiang Cancer Hospital, and written informed consent was obtained from all patients before surgery.

### Clinicopathological factors

Patients who had no smoking in the past 30 days and smoked less than 100 cigarettes in their lifetime were considered non-smokers, those reporting no cigarettes in the past 30 days but having smoked more than 100 cigarettes in their lifetime were regarded as former smokers, and those who smoked at least one day in the past 30 days were considered current smokers [24]. The BMI was calculated by weight (kg) / height (m)^2^, and was grouped according to the following categories: < 18.5 kg/m^2^, ≥ 18.5 to < 24.0 kg/m^2^, ≥ 24.0 to < 28.0 kg/m^2^, ≥ 28.0 kg/m^2^ [25]. The histological type and tumor grade were determined according to the classification criteria for lung tumors of the WHO [26]. The extent of the tumor was determined based on the 7th TNM staging system recommended by the IASLC [27].

The serum CRP, serum albumin, neutrophil count, lymphocyte count and platelet count were all measured preoperatively. The value of serum CPR was determined by latex particle-enhanced immunoturbidimetric assay and the value of serum albumin was mensurated using bromocresol green assay. The CAR was defined as dividing serum CRP value by serum albumin value [28]. The GPS and the mGPS were both determined by the CRP and albumin levels. In the GPS, patients with elevated CRP ( > 10 mg/l) and hypoalbuminemia ( < 35 g/l) were allocated a score of 2; patients with one or neither of these abnormalities were allocated a score of 1 or 0 [29]. As for mGPS, patients with both abnormalities of CRP and albumin levels were given a score of 2; patients with abnormality of CRP level only were given a score of 1; and patients with a normal CRP level were given a score of 0 [30].

### Follow-up

All patients were followed carefully every three months for the first year after surgery and every six months for the subsequent years until patients died or lost to follow up. Follow-up evaluation comprised inquiry, physical examination, blood test, enhanced computational tomography for head and chest, and ultrasoundgraphy for abdomen. The follow-up data included time of follow-up, time of recurrence and metastases, metastatic position, DFS, time and cause of death, and OS.

### Statistical analysis

The cutoff values of CAR, NLR and PLR were determined by receiver operating characteristic (ROC) curve analyses. Then continuous variables like CAR, NLR and PLR were transformed to categorical variables according to the cutoff values above. The chi-square test was performed to assess the correlation between the CAR and clinicopathological factors. The survival curves were generated by Kaplan-Meier estimator and the association between CAR and survival was calculated through Cox proportional hazards regression model. All the statistical analyses were performed with SPSS 13.0 for Windows (Chicago, IL, USA), and *p* value ≤ 0.05 in a two-tailed test was considered statistically significant.
